# Magnetic properties of self-organized Co dimer nanolines on Si/Ag(110)

**DOI:** 10.3762/bjnano.6.80

**Published:** 2015-03-19

**Authors:** Lisa Michez, Kai Chen, Fabien Cheynis, Frédéric Leroy, Alain Ranguis, Haik Jamgotchian, Margrit Hanbücken, Laurence Masson

**Affiliations:** 1 Aix Marseille Université, CNRS, CINaM UMR 7325, 13288 Marseille, France; 2 Synchrotron SOLEIL, L’Orme des Merisiers, Saint-Aubin – BP 48, 91192 Gif-sur-Yvette Cedex, France

**Keywords:** nanomagnetism, one-dimensional nanostructures, scanning tunneling microscopy (STM), self-organization, X-ray magnetic circular dichroism (XMCD)

## Abstract

We demonstrate the kinetically controlled growth of one-dimensional Co nanomagnets with a high lateral order on a nanopatterned Ag(110) surface. First, self-organized Si nanoribbons are formed upon submonolayer condensation of Si on the anisotropic Ag(110) surface. Depending on the growth temperature, individual or regular arrays (with a pitch of 2 nm) of Si nanoribbons can be grown. Next, the Si/Ag(110) system is used as a novel one-dimensional Si template to guide the growth of Co dimer nanolines on top of the Si nanoribbons, taking advantage of the fact that the thermally activated process of Co diffusion into the Si layer is efficiently hindered at 220 K. Magnetic characterization of the Co nanolines using X-ray magnetic circular dichroism reveals that the first atomic Co layer directly adsorbed onto the Si nanoribbons presents a weak magnetic response. However, the second Co layer exhibits an enhanced magnetization, strongly suggesting a ferromagnetic ordering with an in-plane easy axis of magnetization, which is perpendicular to the Co nanolines.

## Introduction

In the last fifteen years, bottom-up approaches have provided promising routes for creating a wide range of nanostructures with new magnetic, electronic, photonic or catalytic properties. Such approaches are based on growth phenomena after atoms and molecules are deposited from the vapor phase onto surfaces. Taking advantage of the intrinsic structural properties of atomically well-defined surfaces, the self-ordering of atoms and molecules allows the fabrication of patterns with nanometer dimensions and precise control over the shape, composition and mesoscale organization of the structures formed.

As growth occurs in many cases under non-equilibrium conditions, the resulting structures result from a competition between kinetics and thermodynamics. With respect to metallic nanostructures, the morphology is essentially determined by kinetics and results from a complex balance of many competing processes occurring at the atomic scale. Each of these processes is thermally activated and characterized by an activation energy. By tuning the growth parameters, such as the substrate temperature or the deposition rate during the deposition of matter, atomistic processes can be selectively promoted or hindered. Using a pre-patterned substrate, networks of metastable, metallic nanostructures exhibiting different geometries can be fabricated on metallic substrates by self-organized growth. Self-ordering proceeds by the preferential nucleation of species on regular-spaced surface traps, which can exist as steps [1–2], atomic sites [3], or the combination of both [4–5], chemical species [6] or dislocation networks [7]. In contrast, when molecules are deposited onto surfaces, the growth is more driven by thermodynamics and molecular arrangements are the result of a delicate balance between lateral interactions between molecules and molecule–substrate coupling. Considering the capability of chemical synthesis to create artificial molecules with a potentially large variety of functionalities, supramolecular [8–11] and covalent [12] assemblies with tailor-made properties can be produced by self-assembly. It has also been reported that nanotemplates can be successfully used to form well-ordered molecular arrays [9,13–17]. Finally, the growth of semiconductor nanostructures is an intermediate case where the pattern is governed by the complex interplay between kinetics and thermodynamics.

The last twenty years have seen an unprecedented rise in the interest in magnetic nanostructures. Besides the interest to potential technological applications, such as magnetic field sensors or magnetic data storage, numerous studies have been devoted to fundamental investigations of magnetism at the nanoscale. Since the discovery of the magnetoresistance effect in 1988, this field has been constantly developing novel nanosystems with unusual physical properties, highlighting the need to study structures of low dimensionality for a fundamental understanding of the physics of the magnetic state. Although less developed, the fabrication of nanostructures of true atomic dimension using a bottom-up approach can result in a deeper insight into the fundamental understanding of their intrinsic properties. For instance, the study of surface-supported two-dimensional (2D) and one-dimensional (1D) Co nanostructures has shown that magnetic properties are highly size dependent, due to the low coordination of the atoms of atomic-scale nanostructures [1,18]. For such nanostructures, enhanced magnetic anisotropy energy (MAE) and orbital moment have been evidenced as compared to the bulk material. Concerning 1D nanostructures, additional effects, especially with regards to magnetic anisotropy, are expected, related to their anisotropic shape [1,19–20]. Since metallic substrates are known to strongly influence the magnetic properties of the supported transition metal nanostructures, it appears interesting to also study the growth of such objects on a non-metallic template. We underline that since self-organized growth allows the fabrication of a high-density of nanostructures with a narrow size distribution, this route of nanofabrication opens up the possibility to investigate their properties using either local or macroscopic integration probes.

In this paper, we show how kinetically controlled growth methods allow for the fabrication of identical, highly ordered, 1D, Co nanostructures on a pre-patterned Ag(110) substrate. For the first step, individual Si nanoribbons (NRs) and high-density arrays (5 × 10^6^ cm^−1^) of Si NRs are formed on Ag(110) upon submonolayer condensation of Si at room temperature (RT) and 460 K, respectively. We have recently shown that Co deposition on the Si/Ag(110) system at RT leads to the self-organized growth of Co dimer nanolines on top of the Si NRs, reproducing the 1D pattern of the Si template. This, however, is limited by defects induced by Co incorporation into the Si NRs [21]. In the experiments reported herein, Co was deposited at 220 K to kinetically block this Co incorporation process and obtain long, defect-free, Co nanolines. The first magnetic characterization results of the Co nanolines using X-ray magnetic circular dichroism (XMCD) are reported, revealing that the atomic Co layer directly adsorbed onto the Si nanoribbons presents a weak magnetic response. The second Co layer exhibits an enhanced magnetization, strongly suggesting a ferromagnetic ordering with an in-plane easy axis of magnetization, perpendicular to the Co nanolines.

## Results and Discussion

### Self-organized growth of Si nanoribbons on Ag(110)

Depending on the temperature of the silver substrate (*T*_sub_) during Si deposition, different geometries of 1D Si nanostructures can be grown on the bare Ag(110) substrate, ranging from isolated, ultrathin, Si NRs to massive 1D nanostuctures corresponding to silver surface faceting [22]. All of these nanostructures are perfectly aligned along the 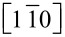 direction of Ag(110). In the following, we will focus on the formation of the Si NRs, which are stable below 550 K and are subsequently used to guide the growth of the Co nanolines.

**Figure 1:**
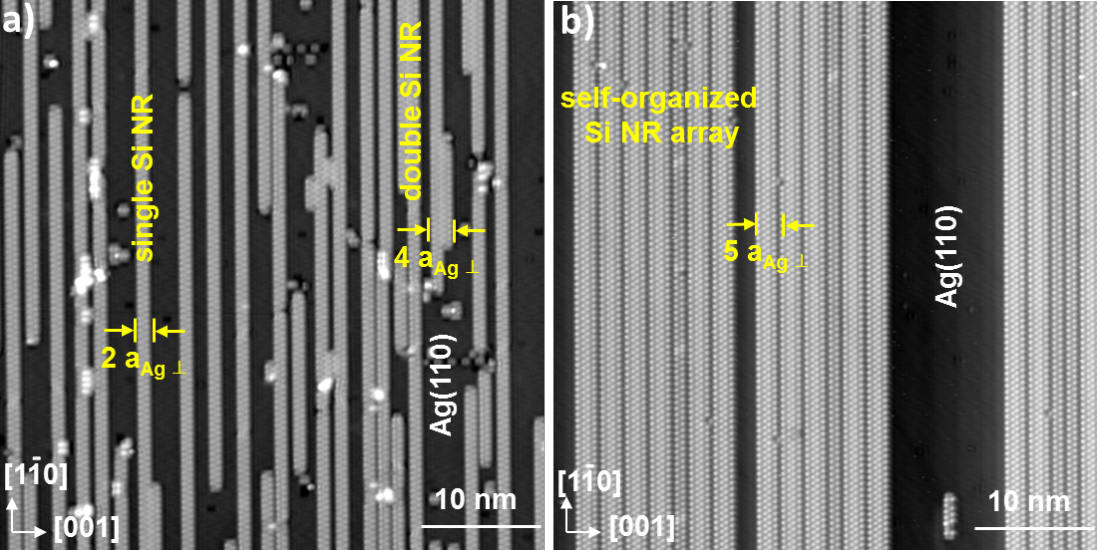
STM images recorded at 77 K at submonolayer Si coverage showing single and double Si nanoribbons (NRs) grown on Ag(110) upon Si deposition at (a) *T*_sub_ = RT, *I* = 300 pA, *V*_sample_ = 1V and (b) *T*_sub_ = 460 K, *I* = 200 pA, *V*_sample_ = 140 mV. The pitch of the Si array is 5 ∙ 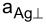 (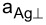 = 0.409 nm, the Ag(110) lattice parameter in the [001] direction).

In their pioneering work, Leandri et al. reported that upon submonolayer Si deposition at RT on the anisotropic Ag(110) surface, isolated Si NRs spontaneously form [23]. As can be viewed in the STM image presented in Figure 1a, the Si NRs are parallel to the atomically dense 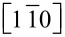 rows of Ag(110) and have been shown to display a 2× periodicity along their edges (2 ∙ 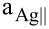 ≈ 0.6 nm) [23]. These NRs, denoted hereafter as single NRs, are composed of two rows of round protrusions [24]. We note that these protrusions are too large to represent individual atoms. We have recently shown that neither STM nor non-contact atomic force microscopy (nc-AFM) probes can straightforwardly resolve the inner atomic structure of the Si NRs [25]. All NRs, varying only in length, present the same width of 2 ∙ 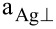 (≈0.8 nm) and the same apparent height: the corrugation measured by STM varies from 50 to 150 pm, depending on tunneling conditions [26]. As shown in Figure 1a, the self-organized Si NRs deposited at RT are randomly distributed on the Ag terraces. Only a few of the grown Si NRs (those corresponding to four row protrusions) present a width of 4 ∙ 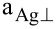 (≈1.6 nm). We emphasize that these NRs differ only in width from the single ones and will be denoted hereafter double NRs. The ratio between double and single NRs increases with *T*_sub_ [24,26]. At *T*_sub_ = 460 K, double NRs are predominantly formed upon Si deposition. These double NRs are self-organized in a regular array with a 5× periodicity in the [001] direction, perpendicular to the NRs (see Figure 1b). At submonolayer coverage, the silver substrate is thus progressively covered upon Si deposition by elongated 2D islands corresponding to the 5 × 2 Si grating. Remarkably, this extremely dense Si NR array has a very low density of defects corresponding to isolated defects or single NRs (more rarely triple NRs (6 ∙ 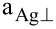)).

At completion of this 5 × 2 arrangement, the entire silver substrate is covered by an ultrathin Si film consisting of a self-organized Si NR array (pitch: 5 ∙ 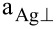 ≈ 2 nm) with a single domain orientation. This structure was confirmed by surface diffraction techniques (low energy electron diffraction, LEED and grazing incidence X-ray diffraction, GIXD) and large scale STM images [24,26]. The sharp spots of the 5 × 2 superstructure displayed in LEED patterns and the narrow GIXD diffraction peaks associated with the 5× periodicity of the superlattice confirm the high structural order of the Si grating. It should be noted that to date, despite the numerous experimental and theoretical investigations on the Si/Ag(110) interface, no reliable atomic structural model for the Si NRs has been proposed.

### Self-organized growth of Co dimer nanolines on Si/Ag(110)

Recent studies have shown that Si NRs grown on Ag(110) can be used as a template for the formation at RT of 1D nanostructures composed of transition metals such as Co [21] or Mn [27]. In both studies, a preferential adsorption on top of the Si NRs with respect to the surrounding uncovered silver areas was reported. Co and Mn are known to easily react with silicon to form silicides. The thermally activated process of Co and Mn diffusion into the Si NRs, which is the first step of the silicide formation, was found to be partially hindered at RT in both systems. This gives rise to the formation of 1D nanostructures, reproducing the 1D pattern of the Si/Ag(110) template.

**Figure 2:**
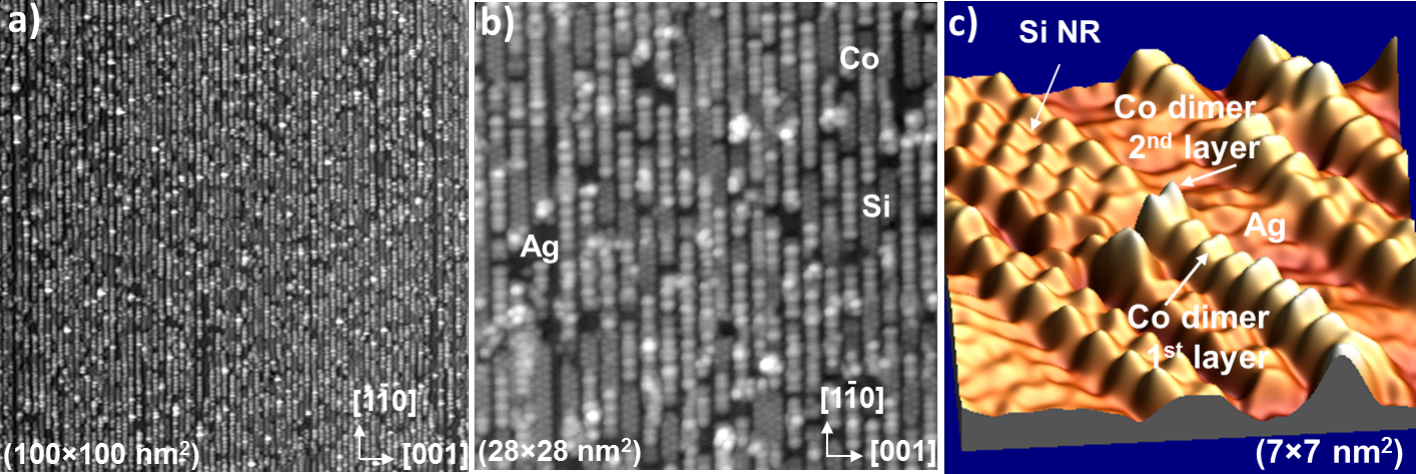
(a,b) STM images at different magnification scales, recorded at 77 K for a Co coverage of approx. 0.6 monolayers, showing the formation of identical and highly ordered Co dimer nanolines on the Si nanoribbon array grown on Ag(110) after Co deposition at 220 K. *I* = 90 pA, *V*_sample_ = −1 V. (c) High-resolution STM image of a Co dimer nanoline grown at RT on a Si nanoribbon (NR). *I* = 1.3 nA, *V*_sample_ = 0.55 V.

First, we reference the results already obtained in our group concerning Co adsorption at RT [21,28–29]. The STM image of Figure 2c shows a typical 1D Co nanostructure formed after Co deposition at RT on isolated Si NRs, partially covering the Ag(110) surface. The grown 1D nanostructures correspond to Co nanolines composed of dimers oriented perpendicular to the axis of the Si NRs. A Co dimer of the second layer can also be observed. The Co–Co distance in a dimer, as measured by STM, is ≈0.4 nm (i.e., ≈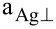) and the distance between two dimers along the nanoline is ≈0.43 nm (i.e., ≈1.5 ∙ 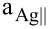). The apparent height of both Co layers is ≈50 pm, suggesting single-atom-thick layers. Interestingly, it has been reported that the Co nanoline growth proceeds in a nearly layer-by-layer growth, reproducing the 1D pattern of the Si template up to five monolayers thick. The width of the Co dimer nanolines is similar to the width of a single Si NR. Co adsorption on double Si NRs leads to the formation of nanolines identical to those observed on single Si NRs, except that most of them are coupled by two on the same double NRs.

Despite the fact that the kinetics of Co diffusion into the Si NRs has been observed to be low at RT at the timescale of our experiments, it has been shown that the length of the Co nanolines is governed by this atomic process of Co in-diffusion rather than the surface diffusion of the adsorbed Co atoms [21]. The incorporation of Co leads to the local destruction of the Si NRs, leaving bare Ag(110) areas. As the activation energy for Co surface diffusion is expected to be lower than that of Co in-diffusion, Co deposition at a lower temperature was performed in the experiments presented here to form longer, defect-free, Co nanolines. The STM images in Figure 2a,b show the formation of identical and highly ordered Co dimer nanolines on the Ag(110) surface completely covered with the Si NR array grown on Ag(110), upon Co deposition at *T*_sub_ = 220 K. The Co coverage is 0.6 ± 0.1 of a monolayer of Co (ML_Co_). 1 ML_Co_ corresponds to the 5 × 2 Si NR array completely covered with Co nanolines and equals 0.6 monolayers (ML) in silver (110) surface atom density. It can be observed that only few, bare silver areas remain, suggesting that the process of Co incorporation into the Si NRs is efficiently blocked at this temperature. In the following section, magnetic characterization of such assemblies of Co nanolines using XMCD is reported.

### Magnetic characterization of the Co dimer nanolines

**Figure 3:**
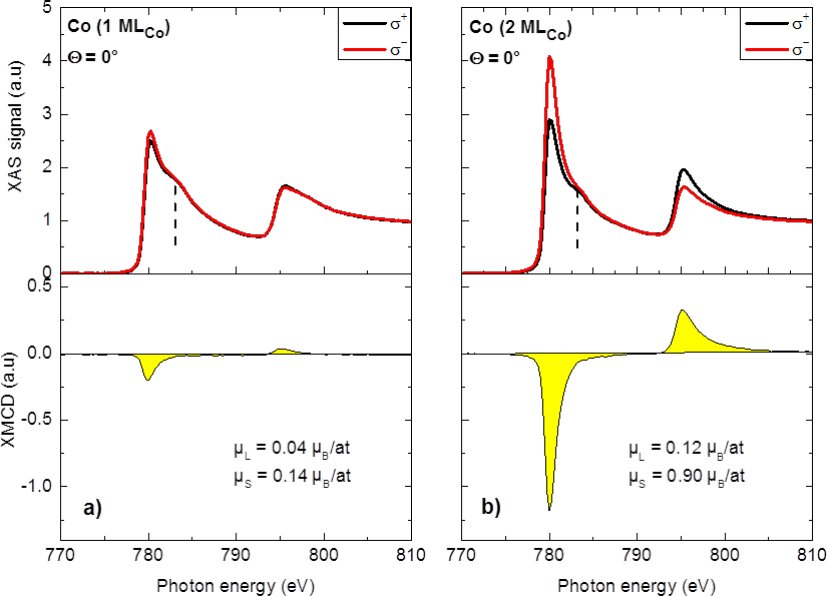
XAS spectra taken at normal incidence (Θ = 0°) for both helicities (σ^+^ and σ^−^) at 4 K with a magnetic field of 6 T and corresponding XMCD signals for (a) 1 ML_Co_ and (b) 2 ML_Co_ on Si/Ag(110). Orbital and spin magnetic moments in both structures were determined by applying the sum rules.

X-ray absorption spectroscopy (XAS) spectra were recorded at normal incidence in a magnetic field of 6 T for parallel (σ^+^) and antiparallel (σ^−^) alignment of the X-ray helicity with respect to the sample magnetization. Magnetic hysteresis measurements at the L_3_ resonance confirm that the sample magnetization is saturated at 6 T. The strong non-magnetic background signal coming from the Ag substrate was subtracted from the Co L_2,3_ XAS spectra presented in this paper. The spectra are also normalized to the incident beam intensity, which is set to zero at the L_3_ pre-edge and to one far above the L_2_ edge. Figure 3a,b shows the XAS spectra for both helicities (upper panel) for ≈1 ML_Co_ and ≈2 ML_Co_, respectively. Two broad absorption resonances are clearly visible at the L_3_ and L_2_ edges. A shoulder peak, indicated in the XAS spectra of Figure 3 by a dotted line, is also present at about 4 eV above the L_3_ edge, located at 779.4 eV. The XAS spectra, which clearly show no trace of cobalt silicides [30–31], are characteristic of metallic Co [32]. Such a lineshape has been seen in numerous structures composed of a thin Co layer grown on a metallic substrate [1,4,32–34] or an insulating support [35]. Although the shoulder at +4 eV from the L_3_ edge can be observed for other Co nanostructures (e.g., an ultrathin 1.25 ML Co film grown on Rh(111) [33] or a superlattice of 0.35 ML 2D Co nanoparticles on Au(788) [4]) this feature is more pronounced in the case of our Co nanolines, especially for low Co coverage. It seems reasonable to exclude the formation of a Co silicide or a Co oxide, since in these cases, a more structured absorption spectrum is expected [30,36–37]. The XAS signal around this energy may be enhanced by the presence of interface states for Co atoms located at the Co/Si interface as suggested by Pong et al. [30]. For both 1 ML_Co_ and 2 ML_Co_, the XAS spectra are similar. However, appreciable differences are present in the XMCD signals reported in the lower panels of Figure 3a,b. The XMCD signal that represents the difference between the XAS spectra for left- and right-handed polarized light gives access to the magnetization direction and magnitude of a specific element. According to the magnetic sum rules [38–39], the spin (μ_S_) and orbital (μ_L_) moments can indeed be quantitatively determined. In this work, we have applied the formalism described by Chen et al. [35] in order to evaluate the spin and orbital contributions to the magnetization of the Co nanolines. The number of holes in the Co 3d band is estimated to be 2.5, which corresponds to the average theoretical value for bulk Co [40–41]. Note that a similar value of 2.4 has been found for the case of Co adatoms on Pt(111) [18]. For 1 ML_Co_, we obtain a spin moment of 0.14 Bohr magneton (μ_B_) and an orbital moment of 0.04 ∙ μ_B_, values, which are considerably smaller than the bulk values given in [35]. The very low dichroism for 1 ML_Co_ reveals a weak magnetic order in this structure when Co is directly adsorbed on Si. Interestingly, a similar Co coverage grown on metallic substrates exhibits a strong magnetic response [1,19,33]. Our results thus evidence that the ultrathin Si layer decouples the Co nanostructures from the metallic substrate, which leads to a drastic decrease of both the orbital and spin magnetic moments.

**Figure 4:**
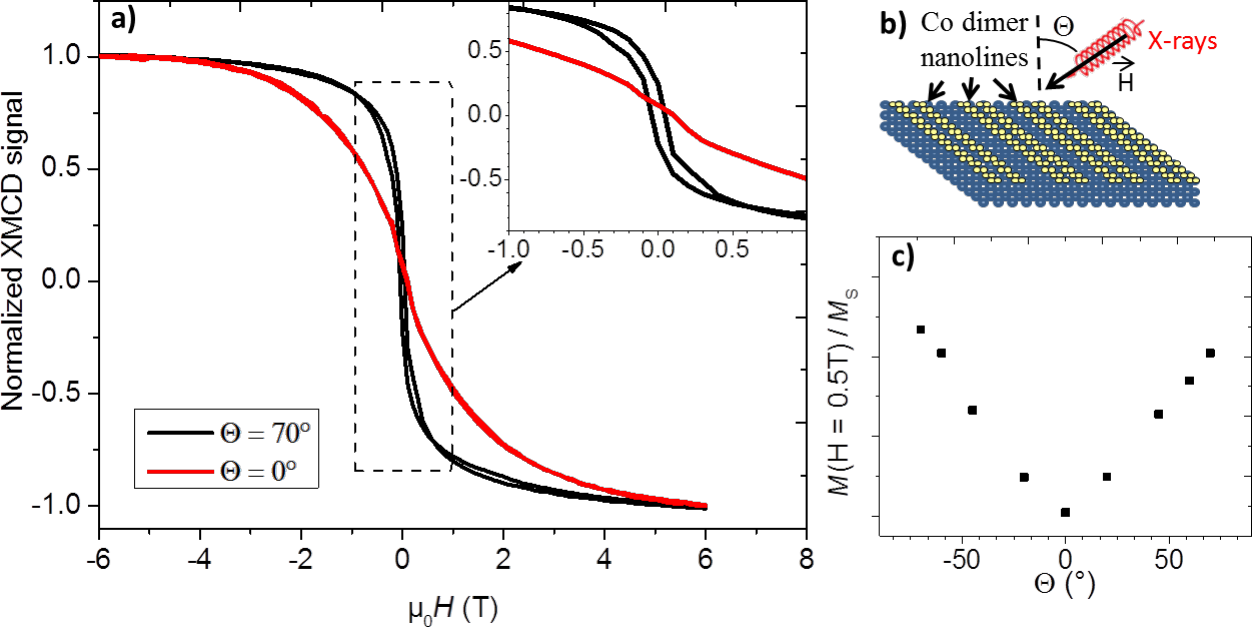
(a) Hysteresis loops of 2 ML_Co_ on Si/Ag(110) measured at 4 K at normal (Θ = 0°) and grazing (Θ = 70°) incidences. The curves have been normalized to their saturation value. (b) Schematic representation of the measurement configuration: incident light and magnetic field are parallel and form an angle Θ with the surface normal in the plane perpendicular to the Co nanolines. (c) Variation of the magnetization at 0.5 T normalized to the saturation magnetization (*M*_S_) as a function of the incidence angle, Θ.

The deposition of a second ML_Co_ leads to a strong enhancement of the XMCD signal. The spin and orbital moments of the ultrathin 2 ML_Co_ film derived from our measurements are 0.90 ∙ µ_B_ and 0.12 ∙ µ_B_, respectively. Taking into account the values found for 1 ML_Co_ and considering that they remain the same in the first layer of the 2 ML_Co_ film, the moments of the Co atoms in the second layer can be estimated as µ_S_ = 1.66 ∙ µ_B_ and µ_L_ = 0.20 ∙ µ_B_. These values, which are close to those of the bulk material (µ_S_ = 1.55 ∙ µ_B_ and µ_L_ = 0.153 ∙ µ_B_) [35], strongly suggest a ferromagnetic ordering. This structure is therefore used to study the magnetic anisotropy in the Co nanolines. The hysteresis loops, obtained from the XMCD signal, were recorded at 4 K for different angles Θ varying from normal incidence (Θ = 0°) to grazing incidence (Θ = 70°) using the measurement geometry presented in Figure 4b. Note that at grazing incidence, the magnetic field is oriented perpendicularly to the Co lines. The hysteresis loops for the two extreme configurations (Θ = 0° and 70°) are presented in Figure 4a and the details of the zero-field region show an opening in the *M–H* curve recorded at Θ = 70°. The square shape of the magnetization curve confirms the presence of significant exchange coupling in the Co film. The angular dependence of the magnetization measured at 0.5 T and normalized to the saturation value is plotted in Figure 4c. The results clearly evidence the presence of an in-plane easy axis of magnetization, perpendicular to the Co nanolines (i.e., along the Co dimer direction). Theoretical [20] and experimental [42] studies related to the 1D Co nanostructures deposited on metallic substrates revealed that the easy axis of magnetization considerably depends on the transverse width of the wires and on the interaction with the substrate. In both cases, an easy axis of magnetization perpendicular to the wires is reported for two-atom-wide wires, in-plane in the case of Co deposited on Pd(110) [20], and with an out-of-plane component for Co bi-chains decorating the steps of the Pt(997) surface [42]. Although the interaction with the underlying layer is expected to be different in our system, our results are consistent with these reported findings. However, our system differs in MAE, which can be estimated from the hysteresis curves [33]. Bearing in mind the very weak dichroic signal recorded for the 1 ML_Co_ deposit, for this calculation, we consider that only the second Co layer contributes to the *M–H* curve. The total magnetic moment has been taken as the sum of both the spin and orbital moments in the second Co layer, which gives 1.86 ∙ µ_B_ per atom. The MAE can be derived from the hysteresis curves displayed in Figure 4a using Equation 2 in [33]. We obtain an in-plane MAE of 0.07 meV per Co atom. This value is small compared to the large out-of-plane anisotropy of Co bi-chains on Pt(997) [42] and to the in-plane anisotropy of Co bi-atomic chains grown on Pd(110) [20]. However, a study of the magnetization angular dependence in the surface plane is required in order to fully characterize the anisotropy of our system and understand its origin.

## Conclusion

In this work, we demonstrated that by tuning the temperature of the silver substrate during Co deposition, the nanopatterned Ag(110) surface consisting of a regular array of Si nanoribbons can be used to guide the self-organized growth of identical Co dimer nanolines with a high lateral order. XMCD measurements revealed that the proximity of the Si template does not affect the metallic character of the Co nanostructures. However, the magnetic properties of the Co nanolines are considerably reduced for low Co coverage when Co is directly adsorbed on Si. The study of the magnetization angle dependence evidences the presence of an in-plane easy axis of magnetization perpendicular to the Co nanolines (i.e., along the Co dimer direction). Another in-plane anisotropy (for instance, along the nanolines) is not excluded, but its demonstration requires further measurements. We stress that due to the presence of a magnetic Co–Si dead layer on the Si template, an efficient decoupling of the Co nanostructures from the metallic silver substrate can be achieved for the upper Co layers, allowing for the characterization of their intrinsic properties.

## Experimental

All experiments were performed in situ in ultra high vacuum (UHV, base pressure, 10^−10^ Torr). The STM images and LEED patterns were recorded at the CINaM in Marseille using an Omicron Nanotechnology STM, working at 77 K and RT. XMCD experiments were performed at the DEIMOS [43] beamline at the French national synchrotron facility (SOLEIL), which operates in the soft X-ray range. XAS was performed in total electron yield mode at the Co L_2,3_ edges. The spectra were recorded at 4 K, under a variable magnetic field of up to 6 T, collinear with the incident X-ray direction. To probe the magnetic anisotropy, the sample was rotated with respect to the magnetic field by an angle Θ, where Θ is the angle between the surface normal and the light beam ranging from 0° (normal incidence) to 70° (grazing incidence), as represented in Figure 4b. The Co/Si/Ag(110) system was obtained using standard procedures for growth experiments in UHV. The Ag(110) sample was prepared by repeated cycles of Ar^+^ sputtering and annealing at 770 K. Si was evaporated on the silver substrate at two different substrate temperatures (RT and 460 K) from either a thermally heated crucible using a commercial Omicron Nanotechnology e-beam evaporator or a direct current heated piece of silicon wafer kept at 1520 K. The Co was deposited using a Co rod (purity 99.99%) inserted in a commercial Omicron Nanotechnology e-beam evaporator. For XMCD measurements, Co was deposited at 220 K on the silver substrate covered with the Si NR grating. The Co coverages in XMCD experiments have been estimated using combined measurements with Auger electron spectroscopy (AES), XAS at the Co L_3_ edge and STM. All STM images were obtained in the constant current mode. The STM data were processed using WSxM and Gwyddion software. The lattice parameters of Ag(110) are denoted 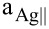 = 0.289 nm in the 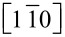 direction and 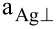 = 0.409 nm in the [001] direction.

## References

[1] Gambardella P, Dallmeyer A, Maiti K, Malagoli M C, Eberhardt W, Kern K, Carbone C (2002). Nature.

[2] Moyen E, Macé M, Agnus G, Fleurence A, Maroutian T, Houzé F, Stupakiewicz A, Masson L, Bartenlian B, Wulfhekel W (2009). Appl Phys Lett.

[3] Repain V, Baudot G, Ellmer H, Rousset S (2002). Europhys Lett.

[4] Weiss N, Cren T, Epple M, Rusponi S, Baudot G, Rohart S, Tejeda A, Repain V, Rousset S, Ohresser P (2005). Phys Rev Lett.

[5] Borca B, Fruchart O, Kritsikis E, Cheynis F, Rousseau A, David P, Meyer C, Toussaint J C (2010). J Magn Magn Mater.

[6] Boishin G, Sun L D, Hohage M, Zeppenfeld P (2002). Surf Sci.

[7] Brune H, Giovannini M, Bromann K, Kern K (1998). Nature.

[8] Yokoyama T, Yokoyama S, Kamikado T, Okuno Y, Mashiko S (2001). Nature.

[9] Theobald J A, Oxtoby N S, Phillips M A, Champness N R, Beton P H (2003). Nature.

[10] Stepanow S, Lingenfelder M, Dmitriev A, Spillmann H, Delvigne E, Lin N, Deng X, Cai C, Barth J V, Kern K (2004). Nat Mater.

[11] Schlickum U, Klappenberger F, Decker R, Zoppellaro G, Klyatskaya S, Ruben M, Kern K, Brune H, Barth J V (2010). J Phys Chem C.

[12] Lackinger M, Heckl W M (2011). J Phys D: Appl Phys.

[13] Weckesser J, De Vita A, Barth J V, Cai C, Kern K (2001). Phys Rev Lett.

[14] Otero R, Naitoh Y, Rosei F, Jiang P, Thostrup P, Gourdon A, Lœgsgaard E, Stensgaard I, Joachim C, Besenbacher F (2004). Angew Chem, Int Ed.

[15] Cañas-Ventura M E, Xiao W, Wasserfallen D, Müllen K, Brune H, Barth J V, Fasel R (2007). Angew Chem, Int Ed.

[16] Berner S, Corso M, Widmer R, Groening O, Laskowski R, Blaha P, Schwarz K, Goriachko A, Over H, Gsell S (2007). Angew Chem, Int Ed.

[17] Aït-Mansour K, Ruffieux P, Gröning P, Fasel R, Gröning O (2009). J Phys Chem C.

[18] Gambardella P, Rusponi S, Veronese M, Dhesi S S, Grazioli C, Dallmeyer A, Cabria I, Zeller R, Dederichs P H, Kern K (2003). Science.

[19] Yan L, Przybylski M, Yafeng L, Wang W H, Barthel J, Kirschner J (2005). Appl Phys Lett.

[20] Félix-Medina R, Dorantes-Dávila J, Pastor G M (2002). New J Phys.

[21] Dettoni F, Sahaf H, Moyen E, Masson L, Hanbücken M (2011). EPL.

[22] Ronci F, Serrano G, Gori P, Cricenti A, Colonna S (2014). Phys Rev B.

[23] Leandri C, Le Lay G, Aufray B, Girardeaux C, Avila J, Dávila M E, Asensio M C, Ottaviani C, Cricenti A (2005). Surf Sci.

[24] Bernard R, Leoni T, Wilson A, Lelaidier T, Sahaf H, Moyen E, Assaud L, Santinacci L, Leroy F, Cheynis F (2013). Phys Rev B.

[25] Leoni T, Bernard R, Ranguis A, Borensztein Y, Prévot G, Masson L (2014). ECS Trans.

[26] Sahaf H, Masson L, Léandri C, Aufray B, Le Lay G, Ronci F (2007). Appl Phys Lett.

[27] De Padova P, Ottaviani C, Ronci F, Colonna S, Olivieri B, Quaresima C, Cricenti A, Dávila M E, Hennies F, Pietzsch A (2013). J Phys: Condens Matter.

[28] Sahaf H, Léandri C, Moyen E, Macé M, Masson L, Hanbücken M (2009). EPL.

[29] Masson L, Sahaf H, Amsalem P, Dettoni F, Moyen E, Koch N, Hanbücken M (2013). Appl Surf Sci.

[30] Pong W F, Chang Y K, Mayanovic R A, Ho G H, Lin H J, Ko S H, Tseng P K, Chen C T, Hiraya A, Watanabe M (1996). Phys Rev B.

[31] Lerch P, Jarlborg T, Codazzi V, Loupias G, Flank A M (1992). Phys Rev B.

[32] Brune H, Gambardella P (2009). Surf Sci.

[33] Lehnert A, Dennler S, Błoński P, Rusponi S, Etzkorn M, Moulas G, Bencok P, Gambardella P, Brune H, Hafner J (2010). Phys Rev B.

[34] Böske T, Clemens W, Carbone C, Eberhardt W (1994). Phys Rev B.

[35] Chen C T, Idzerda Y U, Lin H-J, Smith N V, Meigs G, Chaban E, Ho G H, Pellegrin E, Sette F (1995). Phys Rev Lett.

[36] Regan T J, Ohldag H, Stamm C, Nolting F, Lüning J, Stöhr J, White R L (2001). Phys Rev B.

[37] Gragnaniello L, Agnoli S, Parteder G, Barolo A, Bondino F, Allegretti F, Surnev S, Granozzi G, Netzer F P (2010). Surf Sci.

[38] Thole B T, Carra P, Sette F, van der Laan G (1992). Phys Rev Lett.

[39] Carra P, Thole B T, Altarelli M, Wang X (1993). Phys Rev Lett.

[40] Wu R, Wang D, Freeman A J (1993). Phys Rev Lett.

[41] Guo G Y, Ebert H, Temmerman W M, Durham P J (1994). Phys Rev B.

[42] Gambardella P, Dallmeyer A, Maiti K, Malagoli M C, Rusponi S, Ohresser P, Eberhardt W, Carbone C, Kern K (2004). Phys Rev Lett.

[43] Ohresser P, Otero E, Choueikani F, Chen K, Stanescu S, Deschamps F, Moreno T, Polack F, Lagarde B, Daguerre J-P (2014). Rev Sci Instrum.

